# Peer support for social care in prison: development of guidance and a logic model informed by mixed studies review and co-development workshops

**DOI:** 10.1186/s40352-026-00422-3

**Published:** 2026-07-20

**Authors:** Deborah Buck, Adam O’Neill, Mark Sewell, Eybes Johnson, Noor Butt, Ryan Cowley-Sharp, Paula Harriott, Catherine Robinson, Louise Robinson, Katrina Forsyth

**Affiliations:** 1 University of Manchester, Manchester, United Kingdom; 2 Unlock, Maidstone, United Kingdom; 3 Lancashire and South Cumbria NHS Foundation Trust, Preston, United Kingdom; 4 University of Manchester, Manchester, United Kingdom

## Abstract

Prison peer support schemes can offer social care for people who require help with activities such as self-care, fetching meals, getting around safely, taking part in meaningful activities, and/or forming or maintaining relationships. Little is known about how best to deliver and monitor prison peer support schemes. Peer supporters do not always receive appropriate training or supervision, leaving them and the support recipient vulnerable to exploitation and/or distress or harm. We conducted a mixed studies review to co-develop guidance on peer support schemes for social care in prison and developed an initial programme theory and logic model. We synthesised findings/evidence from stakeholder workshops, six of our previous projects on social care in/on release from prison, and an umbrella review of the literature. The co-developed guidance provides structure for some aspects of the peer support process while allowing for flexibility. We make recommendations about recruitment, training, roles, support plans, and supervision of and support for peer supporters themselves. Better structured/formalised processes which espouse a peer-led approach would empower peer supporters to optimise their capacity to be the “eyes and ears on the ground” and to become recognised, respected and valued as integral to the running of the prison. In turn, this could provide individuals with a sense of purpose, self-efficacy, and self-confidence. The correct balance between risk-management and allowing some flexibility within the peer support role needs to be determined. Our guidance will be implemented and evaluated in at least one prison and disseminated across England and Wales.

**Supplementary Information:**

The online version contains supplementary material available at https://doi.org/10.1186/s40352-026-00422-3.

## Background – Social care and peer support in prison

Prisons worldwide are increasingly recognised as being in crisis (Council of Europe, 2024; Nudd et al., 2024), with overcrowding, inadequate staffing, and staff burnout contributing to environments that do not meet established standards for health and human dignity (United Nations, 2015; World Health Organization, 2014). The UK is not exempt; these issues are prevalent across much of the adult prison estate in England, Wales, Scotland, and Northern Ireland (Nacro, 2024; Howard Leage for Penal Reform, 2024; Scottish Government, 2024; Forsyth et al., 2023; Prisoners’ Education Trust 2023; Morgan et al., 2022).

Increasing rates of imprisonment, longer sentences, and staff shortages coupled with high turnover, mean that many people living in prison are prevented from fully participating in constructive activities, are less able to access healthcare and other services, and are at increased risk of assault, self-harm and human rights violations (Brierley & Dennehy, 2025; Nacro, 2024; Nudd et al., 2024; Prisoners’ Education Trust, 2023). This is particularly concerning given that many people in prison experience physical or mental disabilities or neurodivergence (Mulligan et al., 2025; Prison Reform Trust, 2022; Her Majesty’s Inspectorate of Probation, 2021; Australian Institute of Health & Welfare, 2019; Williams et al., 2012), in turn rendering them at greater risk of developing or deteriorating social care needs. Social care is a term that generally describes personal care (e.g., support with washing, dressing, or getting out of bed) and other practical forms of help to enable people to be safe, active and meaningfully engaged in their community, to live as independently as possible, and to maintain positive relationships with family and friends (The King's Fund, 2024).

Historically, the identification, assessment and provision of social care needs in prison has been ad hoc with needs often going unidentified, inadequately met, or not met at all (O’Neill et al., 2024a; Forsyth et al., 2021; Scottish Government, 2021; World Health Organization, 2020). The Care Act, which was implemented in England in 2015 (Department of Health, 2014), clarified that the responsibility for assessing and providing for social care needs in prison lies with the local authority in which the prison is situated. However, uncertainty remains about roles and responsibilities under the Care Act and its eligibility criteria have been described as ambiguous and open to interpretation (Buck et al., 2024; Burn et al., 2024). Furthermore, the concept of social care is not well understood among many prison officers and healthcare staff (Buck et al., 2024; Her Majesty’s Inspectorate of Prisons, 2018). Thus, social care provision in prisons across England is for the most part sub-optimal with serious gaps remaining (Her Majesty’s Inspectorate of Prisons, 2018; Roulston et al., 2021; Walton et al., 2023, 2025).

One method that has been adopted to try to address the gap has been to enlist the help of peer supporters (Stewart, 2022; The St. Giles Trust, 2017). Peer support can be seen as a type of “social and emotional support that rests on the core tenets of mutual reciprocity, shared problem-solving and empathy” (Perrin, 2020). In the prison setting, this can be in the form of residents helping others with social care/everyday activities that they struggle to manage independently, for example fetching meals, keeping their cells clean and tidy, or getting around the prison safely including attending meaningful activities, socialising, and accessing facilities/services. It is important to clarify that peer supporters are not permitted to provide intimate personal care (National Offender Management Service, 2015).

Provision of peer support can potentially benefit not only the recipient, but also the person providing the support, prison staff, and/or the prison overall. For example, the person providing the support may learn new, transferable skills, improve their self-esteem, sense of responsibility, and increased empathy (House of Commons Justice Committee, 2020; Lee et al., 2019; Lennox et al., 2021; Prisons & Probation Ombudsman, 2016; Walton et al., 2024), and may be less likely to re-offend once released from prison (Lee et al., 2019). Benefits of peer support schemes for prison staff include less pressure on their time/reduced workload (Lee et al., 2016; Walton et al., 2024). These schemes may also have a positive, transformative effect upon the prison overall (Lee et al., 2019) including improved communication between people living in prison and healthcare (Lennox et al., 2021) or improved staff–prisoner relationships (Walton et al., 2024). It is not clear how cost-effective these schemes are (Bagnall et al., 2015; Walton et al., 2024, 2025), and indeed, the evidence base generally is small and tentative, and concerns remain regarding potential harms.

While peer delivery schemes exist in some prisons, there is often a lack of formal training or supervision (Walton et al., 2025; Gavin et al., 2025; Buck et al., 2024; O’Neill et al., 2024a; Davies et al., 2023), and peers may at times feel they have no choice but to provide support inappropriately in place of formal support, e.g., for intimate care (Forsyth et al., 2020; Stewart, 2021; Tucker et al., 2018; Turner & Peacock, 2017). There is also concern that peer support schemes are being rapidly introduced across some prisons seemingly without reference to any evidence-base (Bagnall et al., 2015; Lenkens et al., 2023; Tucker et al., 2018; Walton et al., 2025). Inadequate training, monitoring and evaluation exposes those delivering and receiving peer support to potential risk, including exploitation (Nixon, 2019; Stewart & Edmond, 2017; Walton et al., 2024), and/or burnout (Lee et al., 2019; Perrin, 2020).

Evidence from our recent and ongoing studies, while not specifically focused on peer support, nonetheless revealed that peer supporters were a key component of prison social care provision (Buck et al., 2024, Buck et al., 2025; Hargreaves et al., 2024; Tucker et al., 2018, Tucker et al., 2024; Forsyth et al., 2020; O’Neill et al., 2024b O’Neill et al., 2025). While this was a valuable and important insight, this evidence had not been synthesised in a holistic, systematic fashion or disseminated fully to relevant practitioners, policy makers, or people working or living in prison. Consequently, we secured funding to conduct a ‘pathway to impact’ study, to maximise impact by bringing these findings together. Thus, the present study aimed to develop initial guidance based on our aforementioned work, together with findings from recent literature reviews and the experiences and expert opinion of key stakeholders.

### Research question

What do the evidence and insights from our previous and ongoing work, an umbrella review, and stakeholder workshops tell us about peer support schemes for social care in prison, and how they can be best implemented?

#### Objectives

- To combine evidence from an umbrella review of the literature, stakeholder co-development workshops, and the research team’s previous and ongoing work regarding the use of peer supporters to assist people in prison with social care needs.
- To co-develop good practice guidance for peer support for social care in prison and disseminate nationally.
- To develop a logic model which could help inform the design of future interventions and evaluations.

## Mixed studies review and co-development workshops

### Mixed studies review of our previous work

A mixed studies review involves combining findings from studies with diverse designs and provides a rich and highly practical understanding of complex interventions (Pluye & Hong, 2014). We adopted a mixed studies review approach by first reviewing and synthesising data from six of our previous research projects which had examined health and social care in prison or on release from prison. These projects involved a variety of methods and data sources (interviews, questionnaire surveys, workshops, a scoping review, a realist-informed review, and logic models). The findings from the following studies/programmes of work (some of which are published, others submitted or in preparation) were included:

•Empowered Together (Buck et al, 2024). Involved development of an initial programme theory for social care in prisons, which adopted a realist informed approach.

•Social Care on Release from Prison (SCOR). Involved a scoping review (Tucker et al, 2024), a survey of local authorities (Hargreaves et al, 2024), and qualitative interviews about usual practice and ‘best practice’.

•Dementia and Mild Cognitive Impairment in Prison (DECISION). Involved development of a care pathway and training package (Forsyth et al, 2020), and production of an initial programme theory and logic model.

•Social Care in Prison (SCIP). Involved a survey of local authorities (Tucker et al, 2018).

•‘Low-level’ social care needs in prison (LOSCIP). Involved a scoping review (Buck et al, 2025), and qualitative interviews with people living or working in prison.

•Women’s social care needs in prison (WOSCIP). Qualitative interviews with people living or working in the female prison estate.

### Umbrella review

We conducted an umbrella review to explore the literature on peer support for social care in prison. Umbrella reviews involve synthesising evidence from multiple literature reviews on a specific topic (Gates et al., 2022). Using PubMed, we ran a search in April 2025. We included any review which covered peer support for social care in prison, whether this was the focus of the review or not. The search strategy is provided in Table 1.

**Table 1:** Search strategy

Search ran in PubMed, 16 April 2025
((("peer support*"[Title/Abstract] OR "peer care*"[Title/Abstract] OR buddy[Title/Abstract] OR buddies[Title/Abstract] OR mentor*[Title/Abstract]) AND ("social care"[Title/Abstract] OR "social support"[Title/Abstract])) AND (prison*[Title/Abstract] OR jail*[Title/Abstract])) AND ("systematic review"[Title/Abstract] OR "scoping review"[Title/Abstract] OR "literature review"[Title/Abstract] OR "integrative review"[Title/Abstract])

The search identified three relevant reviews (Lee et al., 2019; Stewart & Edmond, 2017; Walton et al., 2024). Only one of these (Stewart & Edmond, 2017) focussed on peer support specifically for social care in prison. Tellingly, only seven studies met the inclusion criteria, reflecting the dearth of research in this area. The Walton et al (2024) scoping review covered all forms of peer support in prison, not just for social care. While 70 studies were included, the most frequent types of support involved emotional and peer education, while eight involved social care support. Lee et al (2019) conducted a systematic integrative review of programmes addressing the social care needs of older adults in prison. This included 29 papers, eleven of which referred to the involvement of peer support for social care in hospice programmes, and three in ‘structured’ programmes. All three reviews had an international focus (i.e., did not limit to any geographical region). Other key characteristics of the three reviews are provided in Table 2.

**Table 2:** Characteristics of the reviews included in the umbrella review

			**Whether reviews reported on the following issues relating to peer support schemes in prison:**				
**Author, year**	**No. of studies**	**Type of peer support**	**Identifying/Recruiting**	**Training**	**Supervision/support**	**Benefits**	**Challenges/disadvantages**
Walton et al., 2024	70	All (health, emotional, peer education, social care)	Yes	Yes	Yes	Yes	Yes
Lee et al., 2019	29	Programmes addressing the social care needs of older adults in prison (some of which included peer support)	No	Yes	Yes	Yes	Yes
Stewart & Edmond, 2017	7	Peer social care to older prisoners	Yes^a^	Yes	Yes	Yes	Yes

^a^In terms of recommendations

### Co-development workshops

In addition, we facilitated six online meetings/co-development workshops which included a priority setting workshop and a development workshop. In the priority setting workshop, we used a nominal focus group approach to ascertain priorities and key areas for the practice guidance. The nominal group technique is a flexible, iterative focus group method which can be used to explore and reach consensus on a given topic (Thier & Mason, 2018). During the development workshop we refined the guidance to inform the construction of a logic model. A full list of the co-development sessions is provided in Table 3.

**Table 3:** Details of online co-development workshops

Workshop	Mix of attendees	Details/purpose
Introductory workshop	Two experts by experience (EBEs), three research staff, a member of the Prison Reform Trust, and a Director of Social Work	This informal online workshop was held to enable the EBEs and other key team members to meet each other and to begin to explore, from the experts’ perspective, what the main considerations might be when aiming to develop the good practice guidance
Planning meeting	Two EBEs, two research staff, and a member of the Prison Reform Trust	This meeting was held online to prepare for an upcoming priority setting workshop with a wider group of stakeholders
Priority setting workshop	Four EBEs, four research staff, two members of the Prison Reform Trust, a HC governor, a Director of Social Work and four other social work staff	We presented initial findings of the mixed studies review during this workshop. We used a nominal focus group approach to ascertain priorities for the good practice guidance and to ascertain gaps in evidence
EBE validation workshop	Two EBEs and three research staff	Here we focused on the issues that the EBEs had raised in previous meetings, to ensure their expertise has been accurately represented in the notes and to check whether there was anything further they would like to add
Co-development workshop—guidance refinement	Three EBEs, a member of the Prison Reform Trust, a Director of Social Work, two other social work staff, and two research staff	During this workshop discussion focused on refining an updated draft of the practice guidance and informing a logic model
Co-production workshop	Two EBEs, a member of the Prison Reform Trust, and one researcher	This workshop focused on the production of an easy read version of the guidance/summary for publication in Inside Times

### Data synthesis

The method used to consolidate findings from the three strands (workshops, umbrella review, and our previous work) was based on the technique of thematic synthesis of mixed methods (Whittemore & Knafl, 2005). This included data reduction and data display in tables to synthesise findings from the three strands and enabled us to identify key themes to be considered for inclusion in the guidance and logic model.

## Draft guidance: peer support for social care in prison

This section offers a narrative summary of the main elements of the guidance, based on the review of our own work, the umbrella review, and stakeholder workshops. An outline of its overall structure is provided in Table 4.

**Table 4:** Practice guidance for social care peer support schemes in prison

**Recruitment of peer supporters**
Formal job advertisement, vacancy, and application procedures
Formal vetting/recruitment processes
Formal job descriptions
**Training for peer supporters**
Formal training courses (e.g., as provided by RECOOP or a similar third sector organisation)
Ongoing, 'on the job' training
Upskilling
General awareness-raising among the prison population about inclusion, diversity, the ageing process, and dementia
**The role of peer supporters**
Raise awareness about peer support schemes and roles among all prison health, social care, and custodial staff
**Peer support plans**
Co-development of peer support plans for everyone receiving peer support Regular co-review of peer support plans
**Support and supervision for peer supporters**
Supervisor determined by local security strategy
One-to-one review sessions
Peer mentoring groups
Peer support coordinators (‘elevated’ peers)
Governance and monitoring processes
Training for supervisors
Regular meetings between supervisor and support recipient

An overview of key themes from the workshops, mixed studies review, and umbrella review are provided in Supplementary Table S1, which includes the source(s) pertinent to each item/theme. The narrative below is presented in order of the main sections of the guidance, namely the recruitment, training, and roles of peer supporters; peer support plans; and support for/supervision of peer supporters. We also collated information on the reported benefits and challenges of peer support schemes in prison (Supplementary Table S1).

### Guidance on identification and recruitment of peer supporters

The guidance on recruitment proposes that formal procedures should be in place for advertising peer support roles. Peer supporters may be identified through recommendations from staff or self-application. Potential peer supporters should be risk assessed, including security clearance and assessment of risk to self and others in liaison with Safer Custody, which would include a review of their behaviour in prison. In addition, people in need of social care may request another person living in the prison to be their peer supporter. However, it is vital that the potential peer supporter is fully informed of what their duties would be, that acceptance of the role is not compulsory, and that they receive the same vetting, support and supervision as other peer supporters, as outlined above. Where possible, the peer supporter and the person receiving support should be matched to take account of cultural, religious, communication and support needs.

It is recommended that individuals wishing to take on the role of peer supporter are offered help with preparing job applications and with interview skills if needed. Established, experienced peer supporters could be included on job interview panels. Formal job descriptions specific to individuals’ social care needs should be co-developed where possible, with input from the peer supporter and the individual(s) who will be receiving the support. It will be important to show that peer support schemes are mutually beneficial for the person giving and the person receiving support. The role should offer incentives such as payment/financial help, as well as documentation that could help peer supporters after release from prison, such as a formal certificate/qualification. Potentially, this could be formally linked to accredited courses run by a university, and/or apprenticeships could be developed.

### Guidance on training peer supporters

Appropriate training is considered essential for individuals in peer support roles. The section of the draft guidance recommends the delivery of formal training courses and as well as the provision of “on the job” and “real world” training. Formal training courses should be co-developed with and led by individuals with experience of living in prison as a peer supporter. Hands-on learning should be ongoing and should be peer-led. This could involve shadowing, support, and advice. In addition, regular upskilling should be provided and should be needs-led as determined by the individual’s health and social care needs, and the needs and current level of experience of the peer supporter.

Examples of topics that could be covered in peer supporter training for social care include understanding the role; duty of care; Care Act basics; equality and diversity; working in a person-centred way; communication; advocacy; privacy and dignity; safeguarding procedures; referral pathways; boundaries; roles and responsibilities; health and safety; handling information; cleaning and infection control; assisting someone using a wheelchair; mental health; dementia and learning disabilities; health and healthy ageing.

As well as specific training, the guidance recommends general awareness raising, aimed at everyone living or working in prison. This includes raising awareness not only of issues around various social care needs, but also of the ageing process, and matters relating to equality, diversity and inclusion. In addition, specialised training should be considered for disability and assistance peer supporters, and for those supporting people with dementia.

### Guidance on the role of peer supporters

For peer support schemes in prison to run smoothly, participants and staff need to understand and accept the peer supporter role and its value to the prison community. Confidentiality contracts should be established and adhered to. Peer supporters are sometimes offered a shirt or uniform to denote their job. However, experts by experience noted during co-development workshops that often these are not favoured by those in the role due to their conspicuous nature and because they may seem “too authoritarian”, thereby creating a ‘them and us’ divide and tension among people living in prison. Uniforms should therefore not be compulsory. The offer of a badge, which can be easily worn and removed as the peer supporter deems necessary, may be more favourable.

It will be important to consider the number of individuals that peer supporters are asked to support. This will vary depending on the frequency of support that a peer supporter currently gives to each recipient and their own capacity, needs, and aspirations. Because peer supporters have the shared experience of living in prison, they are often more perceptive to and empathetic of recipients’ social care and support needs, including at times needing a listening ear or a familiar face to socialise with over a game of cards. They are therefore in a unique position to offer support at this companionship level in addition to more practical aspects of support. Hence it is imperative that peer supporters, while having agreed responsibilities and boundaries, are also given the flexibility to support individuals in this way as applicable. Nonetheless, the role should be accepted by all as a legitimate job in prison with equal esteem to other work roles/education programmes.

### Guidance on peer support plans

All people living in prison receiving peer support for social care should have an individualised, co-developed peer support plan. The type and frequency of support provided by peer supporters should be built into recipients’ overall care plans. As stipulated in PSI 17/2015 ‘Prisoners assisting other prisoners’ (National Offender Management Service, 2015), the prison’s local lead for social care must ensure that the appropriate boundaries are identified and explained in care and support plans. The plans should be co-reviewed regularly and updated if any changes are made to the type or frequency of peer support, or if a new peer supporter is recruited to the role.

### Guidance on supervision of peer supporters

In line with PSI 06/2012 ‘Prisoner Employment, Training and Skills’ (National Offender Management Service, 2012), peer supporters must be supervised in accordance with the local security strategy. Thus, the person supervising the peer supporter will vary according to each prison’s security status and who is running the peer support scheme (this could be for example a third sector organisation, the LA, prison healthcare, or suitably trained officers). While there are instances of established peer supporters supervising other peer supporters, we recommend that formal supervision is provided by a member of staff, although experienced peer supporters should be available to provide advice and support (as described in a later section).

Supervision of peer supporters should involve relevant governance and monitoring processes. This should include ensuring peer supporters are not exploited in their role by support recipients or other staff. Appropriate training should be undertaken by the supervisors themselves.

Supervisors should meet weekly with the individual receiving the support, to check that their needs are being met adequately and in the agreed manner. These sessions could also be used to help establish whether the type and level of an individual’s need has changed.

Representatives from the Prison Reform Trust suggested that peer supporters could have an annual review which should be recorded rather than being ad hoc. Similarly, experts with lived experience discussed the issue of nominated staff “keeping a watchful eye” on the peer supporter, along the lines of a Personal Development Review found in typical employment positions. However, they emphasised the importance of ensuring that this would not be based on a “totalitarian system” where the peer would feel “under scrutiny”. Therefore, any such review may be best conducted by a social care lead, a third sector representative, the education team, and/or whoever formally supervises the peer supporter.

As well as receiving appropriate supervision, it is equally important that peer supporters are meaningfully supported in their role.

### Guidance on support for peer supporters

Supervisors should facilitate regular one-to-one reviews to help safeguard against negative impact on peers’ well-being and to ensure they do not become overburdened in their role. In addition, our guidance recommends that peer-led group mentoring sessions/forums are held on at least a monthly basis to enable ongoing shared learning and problem-solving, and to provide mutual support.

Other methods of mitigating against burden and distress include reflective support, with a space for peer supporters to regularly off-load their concerns; and teaching peer supporters how to “compartmentalise” aspects of their role, i.e., awareness sessions on how to “push things aside” once they have finished their duties for the day. Experts by experience have noted that this has helped them with their stress levels and to recognise that it is not a “weakness” for peers to talk about any stress or anxiety they may be feeling.

It is vital that appropriate support is offered to peer supporters should a significant incident befall someone they are supporting, given the potential deep emotional impact this can have. For example, in the event of serious illness or death, peer supporters should be notified sensitively and offered compassionate support. Where available and wanted this could involve, for example, mental health services, prison governors, voluntary groups, chaplaincy and/or peer coordinators.

## The logic model of peer support for social care in prison

Logic models (also known as programme theories or theories of change) are visual representations of how an initiative or intervention is assumed to work (Nuffield Trust, 2020; Munro et al., 2018; Office for Health Improvement and Disparities, 2018). Logic models usually focus on a causal chain along the lines of:

$$inputs\to outputs \to responses\to outcomes$$

Inputs are the resources required to address the need; outputs are the intended actions/activities; responses (or mechanisms of change) are the hypothesised responses to and learning from the actions/activities; outcomes are the expected changes following the intervention.

Logic models display the assumptions being made about how the proposed combination of inputs and actions will generate the expected change. Assumptions can be based on previous evidence and/or stem from local experience and ‘common sense’ (Nuffield Trust, 2020). They help to inform transferability of interventions across different settings and to facilitate understanding that is useful to decision makers (Skivington et al., 2021).

**Fig. 1:**
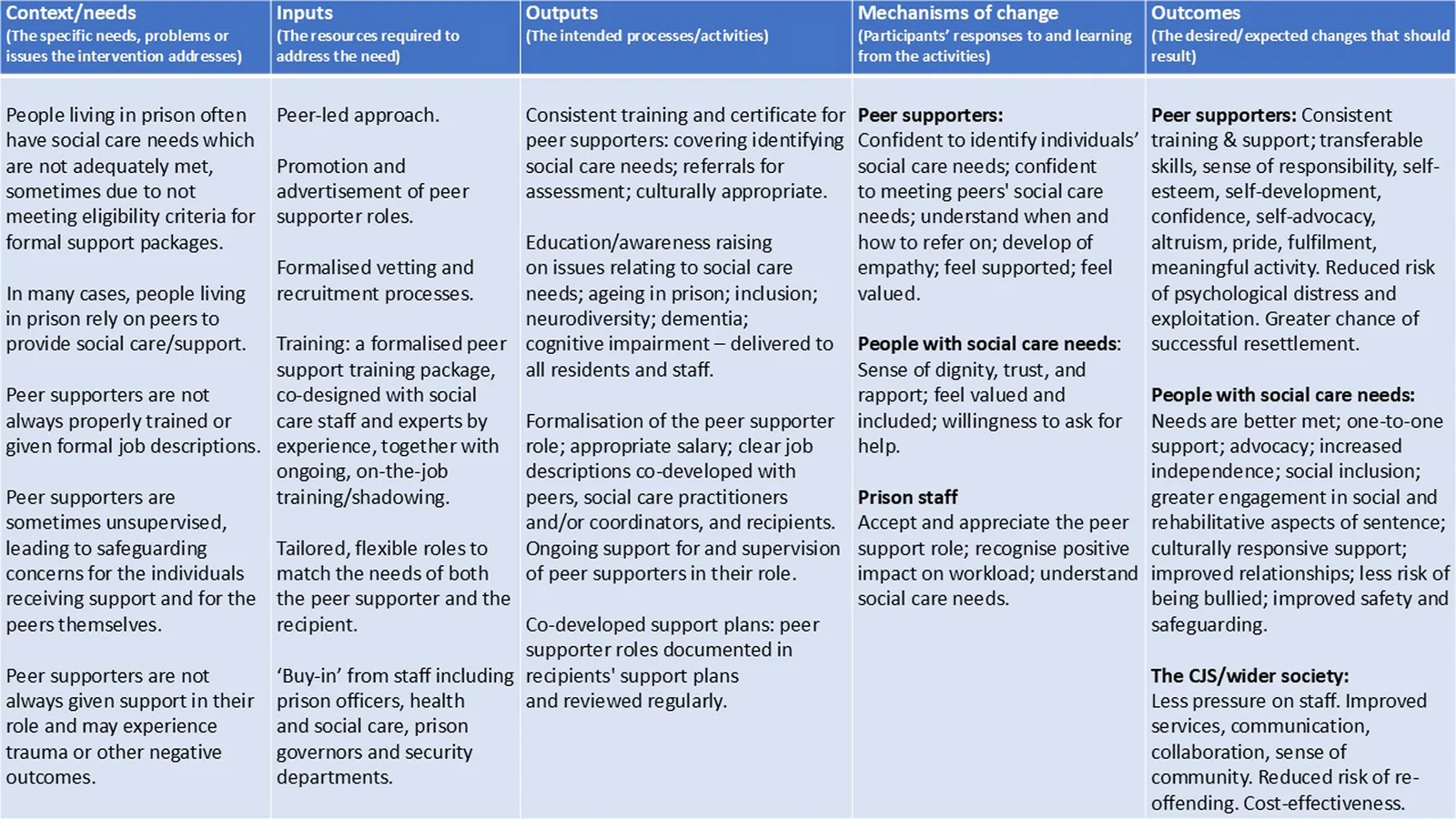
Logic model of peer support schemes for social care in prison

In line with Medical Research Council (MRC) and National Institute for Health Research (NIHR) guidance Skivington et al., 2021), we provide a brief narrative summary of the logic model below to accompany the visual representation portrayed in Fig. 1.

A common theme across this consolidated review was the need for and importance of appropriate training for people living in prison who aspire to the role of peer supporter. Our prior research, though not specifically focussed on peer support, nevertheless repeatedly revealed how integral peer supporters are currently in helping people living in prison to function and participate, and that without this support many would ‘fall through the gap’, leaving them unable to participate in meaningful rehabilitative and personal development activities. Equally, some may not have their basic human needs met, such as being able to regularly access adequate washing facilities, being able to get to the toilet, having access to food, or preserving one’s dignity.

It was clear that training courses for peer supporters need to be tailored, co-developed, and peer led. Co-development should be with practitioners who deliver social care or are responsible for assessing social care needs, with people who have experience of living in prison and have been in a peer support role, and with individuals who themselves have been in receipt of such support.

A particularly important caveat raised by stakeholders was the importance of achieving ‘buy-in’ from all prison staff including prison governors and security departments, as well as external agencies including LAs. Experts with lived experience felt that prison staff should be educated over time about the benefits of peer support schemes, for example by slowly introducing information and guidance rather than suddenly providing “one big document”. Awareness about the presence of social care needs, and the roles of peer supporters, must be raised not only among staff but the prison population in general, so that vulnerable people with social care needs can be supported appropriately, with dignity and respect. Individuals need to be supplied with the relevant information so that they are aware of their basic rights when it comes to social care, and cultural and diversity issues must also be addressed.

Peer supporters have been described by social care staff as being their “eyes and ears” on the ground, including helping to identify people with social care needs. Similarly, peer supporters have described themselves as being “the front line” in terms of being present when support is needed or being particularly perceptive to people’s changing needs. Introducing better structure to the peer supporter role, providing co-developed and ongoing training, rewarding peers with certification and transferable skills, and ensuring adequate supervision will help to ensure that peer support schemes function effectively and safely while optimising an invaluable pool of support.

The logic model hypothesises that adding structure to and formalising peer support schemes in prison will be of benefit not only to the recipients but also to the supporters themselves and the prison community at large including prison officers, and indeed wider society. Provision of appropriate peer support based on mutual respect will enable individuals with social care needs to function, be included, and be as independent as possible while having their basic needs met. Formalised, flexible schemes will furnish peer supporters with meaningful work, transferable skills, increased confidence, and stronger determination to desist from crime once released from prison.

## Discussion

As far as we know, our guidance for peer support in prison is the first co-developed resource, specifically for social care, of its kind. Evidence gleaned from our earlier and ongoing research, together with the umbrella review and insights from key stakeholders including experts by experience, has resulted in a practical guide to good practice. This guidance covers the recruitment and training of peer supporters, offering some degree of structure to the role, development of peer support plans, and supervision of and support for the peer supporters themselves.

There are pockets of innovative practice in peer support for social care in English prisons, details of which were captured in our co-development workshops and other components of the present study. However, many prisons still lack formal, structured systems meaning that peer supporters are often not properly assessed, supervised or supported, and do not receive appropriate types or levels of training. An example of innovative practice, albeit focussed specifically on older people with support needs, is a 14-module ‘Buddy Support Worker Training Programme’, run by a third sector organisation in England named RECOOP. The training programme is built upon the National Care Certificate (occupational standards for health and social care workers). However, it is not clear to what extent the programme has been formally evaluated. The Prisoners’ Education Trust has developed an accredited peer mentoring course which is described as “a valuable first step for people interested in working in social care, youth work and the substance misuse field in or after prison” (Prisoners’ Education Trust 2020). Importantly, although designed as a distance learning course, it was co-written by peer mentors in prison and the people they support. We recommend that any peer support for social care training programmes be co-developed with people with lived experience, be completely in-person rather than online, adopt evidence-based principles and/or, as far as practical, undergo ongoing evaluation.

### Limitations and strengths

While the recommendations within the guidance presented here should be followed as closely as possible, we acknowledge that a degree of flexibility will be required to account for current peer support schemes, where present; local security strategies; and the nature of the physical and social environments within individual establishments. Each of the six studies/programmes of work reviewed as part of guidance development took place in England, and everyone involved in the co-development process lived in or worked for English prisons. However, the umbrella review helped to cast the net wider since none of the included reviews were restricted to England or the UK. Nonetheless, further flexibility may be needed when considering our recommendations in other jurisdictions.

Although some of our previous and ongoing work has centred around the social care needs of people living in the women’s prison estate, the bulk of our work to date has focussed on male prison settings. There are likely to be issues specific to the women’s estate that need to be considered both in terms of social care needs and the peer supporter role.

Most of our previous work upon which the guidance is based examined all adult age groups in prison rather than focussing solely on older people. This helped to ensure that our guidance is at least applicable to all male prisons.

The mixed studies review method provided us with a wide array of germane information to help inform development of the draft guidance. Our previous research did not focus specifically on peer support; thus, some important factors may have been overlooked, However, the umbrella review did focus on peer support, and a key strength has been the meaningful involvement of experts by experience and other stakeholders, in the co-development of the guidance. Their input provided us with a richer and more nuanced understanding of what is needed to really make peer social care support schemes work in the prison setting, what the priorities should be, and how best to foster positive change. Some of the experts by experience had themselves taken on the role of peer supporter in the prison setting. We believe that without this collaboration, any guidance would have been less pertinent and more problematic to transfer into practice.

### Implications for policy and practice

The importance of ‘buy-in’ from all prison staff for establishing peer support schemes, including from governors and security personnel, as well as external agencies, was emphasised throughout the project. Without buy-in, any efforts to introduce or improve peer support schemes and enhance social care provision in prison will fall flat. Moreover, successful sustainable peer support schemes in prison will require sufficient funding and investment. The potential benefits of such systems, not only to recipients and peers but also to staff and the prison community generally, must be made clear to all concerned. By doing so, prison culture could change for the better. In turn, the knock-on effects in terms of better outcomes in and on release from prison should become increasingly apparent.

It is equally important to consider the potential negative consequences of peer supporters crossing the boundary of their role (for example, by providing intimate care, gaining access to confidential information, or being asked to carry out tasks beyond their job description). Such situations could render recipients vulnerable to predatory behaviour, while conversely peer supporters could be at risk of exploitation themselves, and/or experience burnout or trauma. Our co-developed recommendations should help to minimise such undesirable repercussions.

### Future research

The draft guidance will need to be tested in at least one pilot site, and we plan to evaluate and refine the logic model as part of a future intervention in collaboration with people with lived experience of providing or requiring social care in prison. It will be important to determine what factors need to be considered if peer support providers or recipients transfer to a different prison. Equally, future research should explore what the support recipients might need once released from prison, and likewise for peer supporters should they wish to pursue a similar role in the community.

## Conclusions

The draft guidance has been co-developed with experts by experience and other key stakeholders. It represents a practical, free-to-use, standalone resource that can be followed or adapted by individual prison establishments across England and, it is hoped, further afield.

It is imperative that the basic principles of the guidance are followed, particularly that peer support schemes for social care in prison are transparent, structured, and monitored while allowing for some degree of flexibility. This should include formal recruitment processes, risk assessments, job descriptions, renumeration, structured as well as ‘on the job’ training, and ongoing support and supervision. Each aspect of the peer support scheme should be peer-led, thereby bringing authenticity, accessibility and approachability to the role while fostering a sense of shared responsibility and community.

We anticipate that introducing greater structure to and recognition of the peer supporter role will go a long way to optimising this form of support for people with social care needs, make them feel more included, and improve their overall well-being and levels of engagement. This should also “free up” prison officers’ time, and lead to reduced stress and a greater sense of community. Crucially, it should empower peer supporters to enhance their capacity to be the “eyes and ears on the ground” for social care staff, and to become formally recognised, respected and valued as integral to the daily life and running of the prison. Moreover, such achievements are likely to vastly improve peer supporters’ feelings of self-worth and, potentially, their chances of successful resettlement, particularly in terms of education, training and/or employment, and in turn stable accommodation, after release into the community. However, there is an acute need for peer support schemes to be evaluated, to help facilitate the growth of empirical research in this area. It will be important to get the balance right between safeguarding, managing risk, and promoting peer-led schemes in prison.

## Supplementary Information

Supplementary Material 1.

## Data Availability

Data used in developing the guidance is available in Supplementary Table S1.
