# Supplementary material for: Peer support for social care in prison: development of guidance and a logic model informed by mixed studies review and co-development workshops
Source: Health Justice. 2026 Jul 20;14:48. doi: 10.1186/s40352-026-00422-3 (PMC13673686; doi:10.1186/s40352-026-00422-3)
Supplement: Supplementary file 1 [file 40352_2026_422_MOESM1_ESM.docx]

**Supplementary Table S1: Overview of key themes from the mixed studies review, umbrella review, and co-production workshops**

**1. Recruitment of peer supporters in prison**

| **Source of evidence/ insights (see footnote for key)** | **Information on identifying/recruiting peer supporters** |
| --- | --- |
| 1 | Peer supporters are identified through recommendations, self-application, or prisoners requesting another prisoner be their peer supporter. Peer supporters are risk assessed (security vetted and behaviour within prison reviewed). |
| 2c | Identifying potential buddies:  Members from the [third sector] organisation are in the prison each week, for 3 to 5 days a week. They are “interwoven into the fabric of the prison”. They attend various meetings, and on the wings every day meeting existing and potential buddies, and clients.  The organisation asks prison staff to identify any ‘trustworthy’ people who might want to be a buddy; those with “softer qualities because whilst education and training is really important … we're looking for those people that have the softer skills, the personal qualities that have the patience, that are trustworthy” |
| 3 | It is important that peer supporters are appropriately identified and selected.  Where possible, the peer supporter and support receiver should be matched to take account of cultural, religious, communication and support needs. |
| 4b | Prisoners could be screened and trained to provide support to older prisoners with dementia - Du Toit et al 2019 |
| 6 | Participants suggested that people with lived experience should be involved in the recruitment process. |
| 7 | For the peer supporters themselves: have to show there’s a benefit for people doing the role; to show that it is mutually beneficial for person giving and the person receiving support.  Requires approval, acceptance, and drive ‘from the top’. Pointless if governors have buy-in but security don’t. Needs buy-in in from the top and filter down.  Educate officers over time to the benefits of peer support systems; need to educate people/staff slowly rather than just suddenly providing “one big document”. |
| 8a | Peer supporters are risk assessed and go through security and safe custody checks. |
| 10 | In some cases, peers were involved in recruitment as well as coordinating the peer support scheme and training new peer members. |

**2. Training of peer supporters in prison**

| **Source of evidence/**  **insights** | **Information about training** |
| --- | --- |
| 1 | Peer supporters are trained on specific health care needs depending on the individuals’ condition.  More training for disability and assistance peer supporters.  Specific training in one of the women’s prisons included: (Workbook 1) understanding the role; duty of care; equality and diversity; working in a person-centred way; communication and advocacy; privacy and dignity. Workbook 2: health and safety; handling information; cleaning and infection control. Workbook 3: fluids and nutrition; safeguarding adults; assisting someone using a wheelchair; mental health; dementia and learning disabilities; health and healthy ageing.  In another women's prison, training covered: aspects of the Care Act and safeguarding procedures; Care Act basics; requirements; referral pathways; boundaries; roles and responsibilities; what to do if need to raise an issue; basic safeguarding. |
| 2b | C. H. I. O. Care Quality Commission 2018 - Buddies need to be appropriately trained, supported, and supervised. |
| 2c | Selection of buddies: need to be completely qualified and considered a ‘good fit’ for the client (e.g., age group, interests). The organisation will work with and support buddies whose reading and writing is not very good. Might partner them up with a more qualified buddy. 2-tier system to include those with good education and those who struggle with reading/writing, neurodiversity etc., to be as inclusive as possible – the latter have less responsibility, e.g., no advocacy work.  All have to be security checked and cleared.  All buddies are trained to the National healthcare standard of Level 2 in the community.  Training is full time over one week. Then bi-monthly upskilling.  Type and frequency of support is built into care plans.  The organisation supervises, monitors and engages with both the client and the buddy to make sure needs are being met and everybody's happy with the service.  Issues around confidentiality are covered in the training, and there is a confidentiality contract. |
| 3 | Training for peer supporters could include a National Care Certificate equivalent which could be developed, perhaps with the support of a third sector organisation such as RECOOP which offers support and advice in implementing such a scheme for prisons - Munday et al 2017  Peers could act as Healthcare Representatives, with appropriate training and robust procedures in place including adequate planning and management to avoid adverse incidents and abuse of responsibilities, and with a dedicated full-time manager – Lennox et al 2021  Prisons could develop a Buddy Support Service to provide prisoners with mentoring and National Care Certificate equivalent training. This could be developed with the support of a third sector organisation such as RECOOP which offers support and advice in implementing such a scheme for prisons) - Munday et al 2017  Social care in prisons could be delivered by a team of experienced social care staff assisted by a team of trained Care and Support Orderlies (existing prisoners) - Greenwich Prisons Social Care; described in a literature review by Walton et al 2019  ‘If-then’ statement:  If prisons had social care peer support schemes with formal job applications, suitability and security checks, job descriptions, training, supervision, and oversight, then support provided to prisoners could be recorded as part of regular care plan reviews and prisoners would be better supported - Care Quality Commission & HM Inspectorate of Prisons 2018 |
| 4a and 4b | The project involved development of a dementia awareness package for peer supporters in prison. This training package is intended for nominated peer supporters who may provide support (including social care) to people living in prison with dementia. It is designed to help peers look out for signs of dementia/MCI among people living in prison and to provide day-to-day support (not intimate care). As many nominated peer supporters as possible should receive dementia awareness training, led by the Dementia Care Coordinator.  It is important that there is a job description for peer supporters’ roles and that they receive ongoing support and supervision. The impact of the role on the peer supporters’ well-being should be prioritized and continuously reviewed.  The peer supporter training package covers:   - A simple overview of dementia/MCI. - Information about how people living in prison with dementia/MCI can be supported. - What peers can do if they think someone may have dementia/MCI.   The format of the training is a single, face-to-face session lasting no longer than two hours.  UK government guidance relating to boundaries for/supervision of peer supporters should be followed (Prisoners assisting other prisoners: PSI 17/2015). Key points include:   - “All prisoners providing assistance to other prisoners as part of a formal scheme must be appropriately selected, risk assessed, trained, supported and supervised” - “In accordance with PSI 06/2012 Prisoner Employment, Training and Skills, prisoners providing assistance to other prisoners must be provided with appropriate training and support and must be supervised in accordance with the local security strategy.” - “It may also be possible to work with the Skills Funding Agency to commission training to equip prisoners providing assistance to other prisoners from the established education provider. Where generic training for the role is not available, courses may be provided to equip prisoners for particular tasks within it, for example manual handling training for those involved in assisting a prisoner who is unable to move independently.”   The PSI also lists activities which are not permitted, deemed ‘intimate’ care as follows:   - “Assisting with eating and drinking (in the sense of placing food or drink into the mouth, as distinct from other activities to manage and maintain nutrition such as cutting up food and transporting food). - Oral care, including teeth cleaning. - Washing body areas that are usually clothed for privacy and dignity. - Dressing and undressing body areas that are usually clothed for reasons of privacy and dignity. - Toileting support e.g. changing continence pads or sanitary towels. - Assisting an adult with cleaning himself or herself following a soiling or wetting episode.”   “The term personal care is a broader one that applies to tasks that do not require contact with or the exposure of intimate parts of the body. Some examples of personal care include:   - Dressing and undressing that does not involve body areas that are usually clothed for reasons of privacy and decency, for example helping to put on a pair of socks, or a jacket over a shirt. - Maintaining hygiene for bodily areas that are normally exposed. - Providing mental stimulus support for adults that have permanent or temporary mental impairment or diminished mental capacity. - Support with movement or transportation, including moving an appropriately dressed prisoner to the shower or bathroom. - Support with nutritional requirements which do not reach the level of regular assistance with eating and drinking. - Applying make up. - Maintaining personal appearance. - Skin care (of non-intimate areas). - Providing reminders for essential activities like taking medication/ going to the toilet” - “Prisoners must not be permitted to handle, store or administer medication provided to other prisoners. They may, however, (subject to the agreement of the healthcare provider, and in a way that is consistent with medical confidentiality) be involved in providing reminders at the times at which other prisoners are required to take their medication.” - “It is important to be aware of and have sensitivity to cultural differences when agreeing the tasks that a prisoner will perform in each case, and there must be discussions with all parties before a particular task is allocated to a prisoner.” - “In the event that the prisoner for whom assistance is being provided lacks mental capacity, decisions about what is and is not appropriate should be taken on the basis of what would be in the best interests of the prisoner by the prison local lead for social care after consultation with all parties. PSI 15/2015 Adult Social Care sets out the requirements for monitoring arrangements.” The DCC should collaborate with the social care lead. - “Prisoners providing assistance to other prisoners and those who are receiving assistance must be made aware of policies which relate to safeguarding, and in particular how to raise concerns if they witness or experience instances of abuse or neglect.”   Umbrella review conducted as part of 4b  du Toit et al, 2019:   - Training fellow prisoners to support older prisoners/ those with dementia mentioned in 9/35 publications. Schemes need to be closely monitored for predatory behaviour. - "Selection processes (e.g., prisoners with good behaviour for at least 10 years and with no history of cognitive or emotional problems) and prolonged training (up to 12 months) are suggested means to promote the protection of older prisoners.   Brooke et al, 2020:   - Buddy Model initiative (Devon) – prisoners are trained to help other prisoners with SC needs and non-intimate personal care. Training covers CI and dementia, but no robust evaluation has been done. - The Special Needs Program for Inmates with Dementia (California) trained prison officers, nursing staff, and prisoners to support prisoners with dementia.   Treacy et al, 2024:  Examples of good practice include peer support training programmes formulated and delivered by dementia- or older people-specific voluntary organisations. |
| 5 | Need to define peer supporters’ role clearly; formalise it; establish on a prison-by-prison basis the governors’ and staffs’ concepts of who should be a peer supporter and how it should be done. Everyone needs to have ‘buy in’.  *Stakeholders recommended we look at Holly Walton’s work:*  *Rapid evaluation of peer support for social care in prisons in England and Wales). Based on summary of findings which they published online; their full paper is under peer review.*  *Methods: included documentary analysis of HMIP reports, and a mixed methods study.*  *Findings from Holly Walton’s work:*  *Buddy schemes important and valued by staff, peers, and recipients.*  *Benefits include helping recipients maintain independence and participate with prison regime; safeguarding; self/skill development; fulfilment; pride; staff time/workload; improving prison atmosphere. Buddies and recipients generally reported feeling safe. Benefits to wider society.*  *Barriers include low pay, lack of training, regime.*  *Risks include burden, confidentiality, bullying, overstepping boundaries, exploitation of role by staff or recipients.*  *Lots of variation across settings but some good practice (e.g., collaborations between prisons and local authorities, formalised training modules, security vetting).*  *PSI for peer support – guidance may not be consistently met due to lack of training in some establishments.*  *Factors influencing implementation:*  *Service factors – e.g., resources, collaboration with LAs etc.*  *Prison factors – e.g., prison regime; processes and procedures; turnover of buddies.*  *Prisoner factors – e.g., role desirability; respect; reward; recognition; need and attitudes; skills, training, and awareness.*  *Staff factors – e.g., attitudes; skills, training, and awareness.*  *In the site with*out formal peer support, recipients still highlighted social care needs which were supported by informal buddies.  Authors stress the need for appropriate governance processes together with monitoring; Standardisation of services is needed to ensure they are sufficiently resourced and appropriately monitored to mitigate against risks.  Recommendations:  Production and piloting of national guidance for peer supported social care.  Dedicated roles  Appropriate funding  Collaboration between organisations  Formalisation (employment processes, training, and supervision) |
| 6 | Experts by experience:  Training should be delivered by people with lived experience – the importance of them also being ‘a good listener’ and having teaching skills/qualifications was also raised.  Training should include learning how to ‘compartmentalise’, i.e., to not worry about their role once they have finished their tasks (to reduce stress). At the same time, peers should be provided with someone to talk to about their role/stress if they need to. This could include peer-led support groups.  Needs to be incentives, e.g., payment/financial help but also something they can use after release from prison, such as a certificate/qualification  Buy in from governors might be better achieved if people with lived experience met with them and talked about the benefits etc.  Reps from Prison Reform Trust:  Could try to get the training accredited or certified by a college or City and Guilds etc. so that everyone who completed the training, had a certificate  Need to consider how long will the tenure of the position will be.  Peer supporters could have a special t-shirt or uniform and receive extra help towards laundry costs. However, some may not wish to have this as it could make them a target of bullying or harassment.  Views of practitioners in the workshop:  Consider possibility of using work experience and training to demonstrate professionalism, could count towards a formal social care or social work qualification etc. Or as a support worker in the community.  Could be part of a ‘social work apprentice’ placement if people wanted to do a social work degree; could get the training accredited by a University.  Could build up a portfolio that would count towards a training module etc. Could the prison education departments support this, including for people with different learning abilities etc.?  Peer supporters need to know the rights of people in prison with social care needs; know about the Care Act etc. and also what is on offer for those not meeting eligibility criteria  Importance of buy in, joint working and commitment from everyone including LAs. |
| 7 | Negative side is that peers would be easier to ‘single out’ and may be targeted/bullied because of their role. Also extra laundry; extra strains.  The main thing is that they are treated with respect and receive financial benefit.  Instead of a shirt or uniform, peer supporters could wear a simple magnetic badge, and only wear it when needed to if preferred. Uniforms may seem too authoritarian - ‘them and us’ - creates division. |
| 8a | Two tier training:  Tier two peer supporters are fully trained; tier one training is for those who may struggle to do some tasks.  Tier one peer supporters are provided support and training (e.g., education programme) to help them get to tier two if they wish.  Recoop training; Peer supporters complete 14 training modules, each with an exam. They must also complete a 40-question workbook.  Peer supporters are required to sign a contract stating they will honour all the modules of their training.  Peer supporters receive training in autism and dementia; if they are unsure about something they can contact the neurodiversity reps.  Some buddies receive ‘bio training’.  In some establishments bereavement, palliative care and end-of-life care is covered within the training.  Peer supporters are trained to understand the limits of their roles.  In some establishments, peer supporters are trained and supported by Recoop.  Formal training by external organisations can be supplemented by ‘real world’ training delivered by care coordinator prisoners (although recognition that “nothing prepares you for that”). |
| 9 | One local authority had plans to train prisoners on the Prisoners Information Desk so that they could raise awareness of social care needs amongst prisoners. |
| 10 | Peers and staff in two prisons felt the duration and depth of the training was insufficient to support their role, highlighting the need for ongoing training.  Highlighted the need to evaluating training programmes. |

**3. Support for and supervision of peer supporters in prison**

| **Source of evidence/**  **insights** | **Information about support and supervision** |
| --- | --- |
| 1 | Recommend a forum for us [peer supporters] to talk to each other; with an officer there to also talk to. |
| 2b | C. H. I. O. Care Quality Commission 2018 - Buddies need to be risk-assessed |
| 2c | Buddy coordinators (an ‘elevated’ buddy) hold weekly forums with buddies and meet with the clients separately; work in partnership with the social care team and share information; on the wings to observe. Every three months do observations and one to ones - spend a morning or afternoon shadowing the buddy in their duties.  Collect data including what type of support each buddy is providing and how much additional training they have etc. Also collect data on how many people have been able to become independent, and track whether the amount of support the buddy needs to provide can be reduced. Encourage partnership working between buddy and client; helps avoid burnout in buddy. |
| 3 | Recommend introducing group peer mentoring programmes run by people with disabilities (including ex-offenders) – Levy et al 2018 |
| 4b | It is crucial that the well-being of peer supporters is monitored and that they are given ongoing support in their role.  The following prison service instruction (PSI) guidance [16] should be followed:     - “All prisoners providing assistance to other prisoners as part of a formal scheme must be appropriately selected, risk assessed, trained, supported and supervised” - “In accordance with PSI 06/2012 Prisoner Employment, Training and Skills, prisoners providing assistance to other prisoners must be provided with appropriate training and support and must be supervised in accordance with the local security strategy.” - “Local authorities have a duty to prevent the escalation of need, and can be expected to contribute to the training of prisoners providing assistance to other prisoners and to provide support for them as they undertake their work.”   Umbrella review: Ongoing support and supervision were offered/recommended by some prisons, provided mostly by health or social care staff, or charities; informal peer-to-peer support also described. |
| 6 | Peer supporters could have annual reviews as with other jobs, and this governance should be recorded and there is an ongoing record of their activities; and they could get help to develop their CV which could record the training sessions they have attended, work experience etc. |
| 7 | The issue of officers “keeping a watchful eye” – similar to a Personal Development Review in a usual job role. However, important that it is not based on a totalitarian system where the peer feels under scrutiny. |
| 8a | In the event of a death, buddies are notified and asked what support they need by governors. Buddies also receive support through the chaplaincy.  Peer supporters receive support from peer coordinator; if peers have any issues the coordinator will inform the relevant staff and help get the peer supporter the support they need.  Supervision includes ensuring peer supporters are not exploited in their role. |
| 10 | Highlighted the need for risk management and processes to be in place to support and protect peers and recipients from abuse or burden.  Stressed the importance of ongoing supervision. |

**4. Benefits of peer support schemes (including training) in prison**

| **Source of evidence/**  **insights** | **Benefits** |
| --- | --- |
| 1 | Pairing prisoners with peer supporters who speak the same language can help overcome language barriers in prison.  Buddies support less confident prisoners through healthcare processes and advocate for them.  Prisoners may feel more comfortable disclosing certain information to peer workers rather than to staff.  Buddies help new prisoners settle into prison. |
| 2a | “The involvement of trained peer supporters did ... appear to be helpful in identifying people in need of social care and support on release, with such individuals said to: …. have an understanding of the needs of individuals who may require support, and the referral process in that particular prison (Authority ID 950401).” |
| 2c | Privileges, e.g., being allocated to best accommodation; more freedom within the prison.  *“We encourage our buddies then to become community leaders, to become enhanced prisoners. So there's a lot of prison associated benefits that come with being a buddy as well. It kind of fast tracks them.”*  By supporting those in prison who self-isolate or are afraid to leave their cell, can improve their well-being, physical health, mental health and nutrition etc.  Buddies can spend more time with the person than professional staff can.  Buddies are proactive - will let social workers know if feel someone is worried about their release etc. even if not on the SW’s ‘radar’.  Buddies good at promoting independence. E.g., encouraging to help when cleaning cell if they can. This also helps prepare them for release.  Promoting wellbeing is seen as a big focus of the buddy support system.  Peers more likely to notice if someone is struggling. Noticed a huge increase in referrals via buddies.  Those receiving the support from buddies talk about feeling supported.  “... buddies are considered to be trusted prisoners. So they get a lot more respect and admiration … from the staff.” Some do tasks many officers don’t want to do, e.g., cleaning up mess, blood etc. “share the workload” “free up officers’ time” |
| 2d | Peer supporters acted as the “eyes and ears” of social care staff/providers.  Peers were viewed as “invaluable” not only in providing support but also in identifying individuals who might need support on release. |
| 3 | If prisons utilised a 'buddy system' to assist with meeting social care needs of older prisoners, then there may be less pressure on prison staff and increased likelihood of the needs of more prisoners being met – House of Commons Justice Committee 2020 (  If peers could act as Healthcare Representatives, then they could help with some social care needs including supporting access to services, reminding them about appointments and acting as advocates. It could also benefit the peer by increasing self-esteem, self-advocacy, and a sense of responsibility, and could improve services within the prison and improve communication between other prisoners and care providers – Lennox et al 2021  The basic social care needs of prisoners could be more readily met, and the peer supporters would be less likely to feel stressed by their role - Munday et al 2017  Prisoner helper/buddy schemes would help meet some of the (non-personal) social care needs of older prisoners - O’Hara et al 2015  Peer support could be an efficient and cost-effective way of delivering social care within prison - Lee et al 2016 (full)  Peer supporters who receive adequate training and support would gain confidence to provide better informed care, be better able to manage their role, know when to ask for more formal help, and be safeguarded against psychological stressors. Also, prison officers would be more likely to work collaboratively with them - Stewart 2018  Peer supporters who are screened and trained to provide support to older prisoners with dementia would help to ensure person-centred, one-on-one care while providing the peer supporters with valuable rehabilitative skills, and creating a sense of community in the prison environment - Du Toit et al 2019  ‘Trained orderlies’ could assist social care staff in meeting domestic and personal social care needs - Greenwich Prisons Social Care; described in a literature review by Walton et al 2019  (Scotland) While the State cannot shift its duties for care onto other prisoners, it is acknowledged that for many people in prison, caring for or being cared for by a peer is a positive experience – Levy et al 2018  If 'buddy' prisoners were trained and supported throughout their support of other prisoners, then they would feel confident in taking a person-centred approach - Brooke & Jackson 2019  Overall, the programmes were reported to have a generally positive impact, with the transformative effect upon the prison overall and prisoner peer supporters most frequently reported, and peer support particularly commended for the hospice programmes. Peer support provides a positive impact for social care programmes in prison. – Lee et al 2019  If older prisoners had access to buddying systems, then they could build relationships with others and be supported to meet their social care needs – Peacock et al 2019 (  If prisoner carers were appropriately trained, monitored, assessed, and not overly relied upon, then benefits would be seen for the carer (skills, altruism) and the person being cared for (helping with day-to-day activities) - Prisons and Probation Ombudsman 2016 |
| 4b | Training gives younger prisoners transferable skills that may assist with resettlement and crime desistance on release, can facilitate a sense of responsibility, payment, and is linked to diminished bullying of older prisoners.  "The benefits of trained buddies or aides for both prison system and older prisoners with dementia are that they provide one-on-one care and high-quality, lower-cost alternative; facilitate greater levels of choice and provision of culturally responsive peer support; and could promote an increased sense of community among prisoners and between staff and prisoners (Stewart and Lovely, 2017)."  Umbrella review: Reported benefits of dementia training for peers: improved understanding of dementia, relationships, and diagnoses. |
| 8a | Buddies have shared experiences with prisoners and are able to relate to prisoners’ experiences and offer advice.  Buddies are trusted prisoners. If they witness an incident, they will report back to the officers who can then intervene.  Prisoners are more likely to accept the support of their buddies since they can get to know them in a deeper way and learn more about them than other prison staff.  Buddies can intervene immediately and isolate prisoners who are ill, preventing the spread of illness until a member of staff is able to attend.  Having a buddy gives prisoners independence from the prison system.  Buddies can have brief, informal conversations with prisoners to determine how they are feeling/coping on a given day.  Buddies are “the front line”; they are able to notice small changes in prisoners' behaviour as they can move freely around the wing/landing and speak to prisoners; they are usually the first to respond if someone has a fall etc.  Some individuals do not want involvement with social workers, so peer supporters can help with support needs which may otherwise not be met. |
| 8b | Provision of peer support, with appropriate oversight and vetting procedures, may help slow down the development of ‘higher-level’ social care needs. |
| 10 | Improved staff–prisoner relationships.  Increasing capacity and reducing workload for staff.  Personal growth, employment skill development, and motivation for peers.  Support from someone similar, strengthened relationships, and emotional support for recipients.  Prisoner peer supporters working on hospice programmes: rewarding work; a transformative effect on their self-confidence, sense of compassion, self-awareness, and community; increasing opportunities for redemption and rehabilitation; predicted reduction in recidivism; prisoner peer supporters were reported to find the work rewarding.  Assisting people to adapt to prison processes; reducing the effects of institutionalisation.  One study included an evaluation of bespoke and formal social care training in three UK adult male prisons (focusing on social care for older prisoners) and reported that the training enhanced peers’ insight into caregiving. Other stakeholders including prison staff felt it promoted dignity and respect among the prison population. |

**5. Challenges / disadvantages of peer support systems in prison**

| 1 | Peer support systems may only be effective when prisoners are paired with the right buddy.  Lack of structure within some prisons for the peer support system; lack of clearly defined roles means some staff are unaware of peers’ responsibilities.  Some staff see peer supporters as “like a hindrance rather than helpful”.  Lack of training for peer supporters in some establishments; no consistency.  “No oversight and no management at all.”  Lack of support for peer supporters themselves. |
| --- | --- |
| 2b | C. H. I. O. Care Quality Commission 2018:  The authors note not that all prisoners they spoke to as part of the report were comfortable about other prisoners being involved in the screening process due to concerns about confidentiality. |
| 2c | LA staff:  People may manage in custodial setting when have support from peers but can’t manage after being released - importance of making the receiving LA aware of this. Refers to ‘low level’ support from peers as opposed to personal care, e.g., helping to get their meals. People who don't meet the eligibility criteria might get support from a peer – if in the community, that might trigger a care package if person has nobody. Also implies that without the peer support in prison, a care package would be needed. Quote: *“very kind of low level stuff. Where in the community… if you needed help with that and you didn't have anybody, then obviously you know it, it may trigger eligibility. I think … some of the requests the prison may already be meeting and therefore it's kind of felt actually … you're putting in what's adequate enough that it's not really triggering anything else that is needed from adult social care”*  A third sector organisation:  System works very well when there is ‘buy in’ and engagement from the establishment. More difficult in remand prisons due to quick turnover of buddies and clients. Try to ensure that anyone trained in a remand prison is given the opportunity to work as a buddy in their new prison. Opposition from wing staff/officers, particularly the ‘old school’ officers. Younger officers tend to be more ‘clued up’ about inclusion, diversity, neurodiverse rehabilitation etc. Distinction between thinking prison is to punish vs. to rehabilitate. Officers may even prevent buddies carrying out their role. Some prisoners won't admit to having social care needs if they don’t have long left on their sentence because are concerned would ruin any chances of transfer to an open prison before release. |
| 4c | Lack of clarity about peer supporter’s roles, leading to “blurred boundaries” for the supporters and individuals receiving the support. Some peer supporters would go “beyond what is expected” and in some cases do so without receiving any payment for their role.  Relatively “simplistic” recruitment processes.  Lack of clarity about the degree and content of training.  Insufficient or no training, leaving peer supporters feeling ill-prepared. |
| 6 | High turnover of prison officers makes it difficult to maintain buy-in from staff.  Buy-in often comes down to having “the right people” among the establishment’s staff. |
| 7 | The negative side of having a special shirt/uniform for peer supporters is that peers would be easier to ‘single out’ and may be targeted/bullied because of their role. Also, extra laundry; extra strains.  The main thing is that they are treated with respect and receive financial benefit.  Instead of a shirt they could wear a simple magnetic badge and wear it when needed. Uniforms may seem too authoritarian - ‘them and us’ - creates division. |
| 8a | Peer supporters do not receive enough compensation for their work.  Peer supporters are mistrusted by some prisoners and perceived as on the same side of the officers.  Peer supporters may be exploited by prisoners.  Not enough training on dementia for peer supporters and no training on support at end-of-life.  Possible abuse of/stealing medication by peer supporters. However, there is prison and buddy coordinator oversight to ensure this doesn't happen or is dealt with.  Peer supporters are not appreciated by governors.  Peer supporters do not receive enough support. |
| 10 | Challenges include confidentiality, burden, prisoner safety, ensuring the right motivations, the potential for abuse, and queries around who should be allowed to be involved.  Some peer supporters reported burnout, exacerbated by inadequate training and supervision.  Social transformation could be perceived as a threat to the prison authority; training and giving new responsibilities to peers may affect the power balance between prisoners and prison staff and may result in resistance among prison officers. |

**Footnote: Key to sources of evidence/insights**

1 Women’s social care in prison (WOSCIP) (paper in preparation)

2a Social care on release (SCOR): survey of local authorities (Hargreaves et al, 2024)

2b SCOR scoping review (Tucker et al, 2024)

2c SCOR qualitative interviews re: usual practice (paper in preparation)

2d SCOR qualitative interviews re: best practice (paper in preparation)

3 Empowered Together: Programme theory/logic model for social care in prisons and on release (Buck et al, 2024)

4a DECISION (dementia/MCI in prison) - development of a care pathway and training package (Forsyth et al, 2020)

4b DECISION - development of an IPT and logic model (paper submitted)

4c DECISION – qualitative interviews (O’Neill et al, 2025)

5 Initial stakeholder workshop

6 Priority-setting workshop

7 Validation workshop

8a ‘Low-level’ social care in prison (LOSCIP) qualitative interviews (paper in preparation)

8b LOSCIP scoping review (Buck et al, 2025)

9 Social Care in Prison (SCIP) (Tucker et al, 2018)

10 Umbrella review (Walton et al 2024; Lee et al 2019; Stewart & Edmond 2017)

**References**

Buck D et al (2025) ‘Low-Level’ Social Care Needs of Adults in Prison (LOSCIP): A Scoping Review of the UK Literature. Social Sciences. 14(2):112. doi:10.3390/socsci14020112

Buck D et al (2024) Developing an initial programme theory for a model of social care in prisons and on release (empowered together): A realist synthesis approach. Medicine, Science and the Law. 2024;0(0). doi:10.1177/00258024241264762

Forsyth et al (2020) Dementia and mild cognitive impairment in prisoners aged over 50 years in England and Wales: a mixed-methods study. Health Soc Care Deliv Res; 8(27). doi:10.3310/hsdr08270

Hargreaves C et al (2024) Some improvements but a long way to go: a national survey of local authorities on the provision of social care for people released from prison. Health Justice. 2024 Nov 29;12(1):47. doi: 10.1186/s40352-024-00304-6.

Lee C et al (2019) A systematic integrative review of programmes addressing the social care needs of older prisoners. Health Justice, 27,7(1):9

National Offender Management Service (2015) Prisoners assisting other prisoners. Available at:

https://assets.publishing.service.gov.uk/media/5f22eb5c8fa8f57ac440fa52/psi-17-2015-prisoners-assisting-other-prisoners.pdf

O’Neill A et al (2025) Providing appropriate health and social care for people with dementia or mild cognitive impairment in the criminal justice system of England and Wales: a thematic analysis of prisoner and staff interview data. Health Justice. 2025 Jan 20;13(1):5. doi: 10.1186/s40352-024-00313-5.

Stewart W, Edmond N (2017) Prisoner peer caregiving: a literature review. Nurs Stand. 31(32):44-51. doi: 10.7748/ns.2017.e10468

Tucker S et al (2024) Supporting people with social care needs on release from prison: A scoping review. Journal of Long-Term Care, 90–106. 10.31389/jltc.161

Tucker S et al (2018) Social care in prison: emerging practice arrangements consequent upon the introduction of the 2014 Care Act. The British Journal of Social Work, 48(6): 1627–1644. DOI: <https://doi.org/10.1093/bjsw/bcx115>

Walton H et al (2024) Peer support for health, social care, and educational needs in adult prisons: a systematic scoping review. Public Health, 236: 412-421. doi:10.1016/j.puhe.2024.08.002

Walton et al

Summary (full paper was under peer review as of Nov 2023)

https://www.nuffieldtrust.org.uk/sites/default/files/2023-11/NIHR%20RSET%20-%20Peer%20support%20for%20adult%20social%20care%20in%20prisons%20slideset_15112023%20%20%281%29.pdf

Q&A

https://www.nuffieldtrust.org.uk/news-item/peer-support-for-adult-social-care-in-prisons-in-england-and-wales-a-qa-with-dr-holly-walton-and-chris-sherlaw-johnson#:~:text=Across%20England%20and%20Wales's%20prisons,gap%20in%20social%20care%20provision.
